# Effectiveness and Cost Effectiveness of Oral Pre-Exposure Prophylaxis in a Portfolio of Prevention Programs for Injection Drug Users in Mixed HIV Epidemics

**DOI:** 10.1371/journal.pone.0086584

**Published:** 2014-01-28

**Authors:** Sabina S. Alistar, Douglas K. Owens, Margaret L. Brandeau

**Affiliations:** 1 Department of Management Science and Engineering, Stanford University, Stanford, California, United States of America; 2 Veterans Affairs Palo Healthcare System, Palo Alto, California, United States of America; 3 Center for Health Policy/Program on Clinical Outcomes Research, Stanford University, Stanford, California, United States of America; Hopital Bichat Claude Bernard, France

## Abstract

**Background:**

Pre-exposure prophylaxis with oral antiretroviral treatment (oral PrEP) for HIV-uninfected injection drug users (IDUs) is potentially useful in controlling HIV epidemics with a significant injection drug use component. We estimated the effectiveness and cost effectiveness of strategies for using oral PrEP in various combinations with methadone maintenance treatment (MMT) and antiretroviral treatment (ART) in Ukraine, a representative case for mixed HIV epidemics.

**Methods and Findings:**

We developed a dynamic compartmental model of the HIV epidemic in a population of non-IDUs, IDUs who inject opiates, and IDUs in MMT, adding an oral PrEP program (tenofovir/emtricitabine, 49% susceptibility reduction) for uninfected IDUs. We analyzed intervention portfolios consisting of oral PrEP (25% or 50% of uninfected IDUs), MMT (25% of IDUs), and ART (80% of all eligible patients). We measured health care costs, quality-adjusted life years (QALYs), HIV prevalence, HIV infections averted, and incremental cost effectiveness. A combination of PrEP for 50% of IDUs and MMT lowered HIV prevalence the most in both IDUs and the general population. ART combined with MMT and PrEP (50% access) averted the most infections (14,267). For a PrEP cost of $950, the most cost-effective strategy was MMT, at $520/QALY gained versus no intervention. The next most cost-effective strategy consisted of MMT and ART, costing $1,000/QALY gained compared to MMT alone. Further adding PrEP (25% access) was also cost effective by World Health Organization standards, at $1,700/QALY gained. PrEP alone became as cost effective as MMT at a cost of $650, and cost saving at $370 or less.

**Conclusions:**

Oral PrEP for IDUs can be part of an effective and cost-effective strategy to control HIV in regions where injection drug use is a significant driver of the epidemic. Where budgets are limited, focusing on MMT and ART access should be the priority, unless PrEP has low cost.

## Introduction

Recent advances in HIV prevention and treatment have increased hope that the global HIV epidemic can be controlled. New HIV infections have decreased in regions of the world that have traditionally been the source of highest concern, such as countries in sub-Saharan Africa [1]. Despite these encouraging results, in other parts of the world the HIV epidemic continues to grow. In particular, in Eastern Europe where limited prevention measures have been implemented, HIV continues to spread rapidly, fueled by significant levels of injection drug use [1].

Pre-exposure prophylaxis with antiretroviral drugs (PrEP) has garnered significant attention in the past several years, particularly because of its potential to be used by uninfected individuals in key population groups (injection drug users (IDUs), sex workers) to protect themselves when other means of protection (condoms, clean needles, etc.) are unavailable or cannot be used. Several clinical trials have shown oral PrEP to be effective in reducing the chance of HIV infection acquisition. A clinical trial of daily oral tenofovir disoproxil fumarate and emtricitabine (TDF/FTC) among men who have sex with men found that PrEP reduced HIV acquisition by 42% [2]. Among heterosexuals, recent clinical trials found a 67% reduction in HIV acquisition among HIV discordant couples for TDF, a 75% reduction for TDF/FTC [3] and a 62% reduction in HIV acquisition among heterosexually active men and women taking daily oral TDF/FTC [4]. Two other clinical trials among heterosexuals found no reduction in risk of HIV acquisition, likely because of poor adherence [5], [6]. In June 2013, a clinical trial of daily oral TDF for uninfected IDUs in Thailand reported a 49% decrease in HIV acquisition due to PrEP [7]. Shortly thereafter, the US Centers for Disease Control and Prevention (CDC) published interim guidance suggesting that PrEP be considered as one of several prevention options for high-risk IDUs in the US [8].

The role of oral PrEP in HIV epidemics where injection drug use is a significant contributor to the spread of HIV, such as those in Eastern Europe, has not been investigated. We sought to evaluate the cost effectiveness of PrEP for IDUs alone or as part of a portfolio of interventions including methadone maintenance treatment (MMT) for IDUs and antiretroviral treatment (ART) for all infected individuals. MMT, a replacement therapy that substitutes methadone for opioids with the aim of reducing or eliminating drug injection, has been shown to be an effective and cost effective means of reducing HIV spread among IDUs [9]–[12]. We developed a dynamic model of HIV transmission and progression which we used to project the evolution of the epidemic under various combinations of strategies for HIV control: oral PrEP programs for uninfected IDUs, MMT programs for IDUs, and scale-up of ART programs for eligible HIV-infected individuals (including IDUs and non-IDUs). We used data for Ukraine, which has the highest HIV prevalence in Europe, and one of the fastest growing epidemics in the world.

## Methods

### Overview

We extended a previously developed dynamic compartmental model of the HIV epidemic in a population of non-IDUs, IDUs who inject opiates, and IDUs on methadone [9], adding an oral PrEP program for uninfected IDUs (Appendix S1; Figure S1 and Table S1 in Appendix S1). We modeled 1,000,000 individuals aged 15–49 stratified by HIV status and injection drug use. In the model, individuals can acquire HIV through unsafe sexual contacts or injection equipment sharing with HIV-infected individuals. We instantiated the model with data for Ukraine (Table 1; Table S2 in Appendix S1). The model is implemented in Microsoft Excel.

**Table 1:** Key parameter values, ranges, and sources.

Parameter	Value	Range	Source
**Prevalence**			
Initial HIV prevalence IDUs	41.2%	17.3%–70.0%	[14]–[19]
Initial HIV prevalence non-IDUs	0.99%	0.73%–1.16%	Estimated [9], [19]
**Injection behavior**			
Number of injections per year	250	200–300	[9], [21], [22], [55]–[57]
Percent of shared injections	25%	10%–40%	[9], [21], [22], [55]–[57]
**Sexual behavior**			
Number of sexual partners per year – IDUs	4.3	1.5–4.5	[9], [21], [22]
Number of sexual partners per year – non-IDUs	1.3	1–1.8	[9], [21], [22]
Percentage of IDU sexual contacts with other IDUs	45%	20%–70%	[9], [21], [22], [56], [57]
Condom usage rate – IDUs not on MMT or PrEP	40%	20%–60%	[9], [21], [22], [56], [57]
Condom usage rate – IDUs not on MMT but on PrEP	40%	20%–60%	Estimated
Condom usage rate – IDUs on MMT but not PrEP	45%	25%–65%	[9], [21], [22], [56], [57]
Condom usage rate – IDUs on MMT and PrEP	45%	25%–65%	Estimated
Condom usage rate – non-IDUs	45%	30%–70%	[9], [21], [22], [56], [57]
Condom effectiveness	90%	85%–95%	[10], [11], [21], [60]
**Antiretroviral therapy (ART)**			
Access to ART – eligible non-IDUs	22%	7%–11%	[13]
Access to ART – eligible IDUs	2%	0%–5%	Estimated [55], [56], [61]
Access to ART – eligible IDUs on MMT	25%	0%–30%	Estimated [35], [62]
Sexual transmission reduction if on ART	96%	50%–99%	[21], [23], [30], [31]
Needle sharing transmission reduction if on ART	50%	10%–90%	Estimated [9], [21], [31]
**Methadone maintenance treatment (MMT)**			
Percent decrease in injection equipment sharing if on MMT	85%	60%–99%	[10], [11], [35], [36], [62]
MMT retention, 6 months	75%	50%–90%	[35], [62]
Percentage MMT “graduation”	5%	1%–7%	[35], [62]
**Pre-exposure prophylaxis (PrEP)**			
Percent change in risky injections due to PrEP	0%	−20%−20%	[38]–[40]
Percent change in risky sexual contacts due to PrEP	0%	−20%−20%	[38]–[40]
Sexual transmission reduction if on PrEP	49%	10%−72%	[7]
Needle sharing transmission reduction if on PrEP	49%	10%−72%	[7]
**Annual costs (US$)**			
Non-HIV medical care	311	200–450	[43]
HIV care	1200	800–1600	Estimated [14]
ART - IDUs not on MMT (including IDU services)	950	750–2500	[9], [21], [32]–[34]
ART - IDUs on MMT (including IDU services)	750	550–2300	[9], [21], [32]–[34]
ART - non-IDUs	450	250–2000	[9], [21], [32]–[34]
MMT (including counseling services)	368	200–500	[9], [21], [37]
PrEP (including counseling services)	950	100–1500	Estimated

IDU = injection drug user, ART = antiretroviral therapy, MMT = methadone maintenance treatment, PrEP = pre-exposure prophylaxi.

Under the status quo, approximately 22% of eligible HIV-infected individuals receive ART [13], virtually no IDUs receive MMT [14], [15], and no IDUs receive PrEP. We considered the following levels of intervention scale up, alone and in combination: introduction of PrEP for 25% or 50% of uninfected IDUs (representing a moderate and relatively high level of PrEP, respectively); scale up of MMT to 25% of IDUs; and scale up of ART to 80% of eligible HIV-infected individuals, including both IDUs and non-IDUs (which we will refer to as “universal ART coverage”). We assumed that the interventions would be fully implemented at the start of the modeled time horizon: for example, for the case of 25% PrEP, 25% of IDUs received PrEP at the start of the time horizon and this fraction was held constant throughout the modeled time horizon. We measured health care costs, quality-adjusted life years (QALYs), HIV prevalence, HIV infections averted, and incremental cost effectiveness.

### Model Structure and Transitions

We segmented the population into 20 compartments distinguished by HIV status (uninfected, early stage HIV (CD4>350 cells/µl), late stage HIV (CD4 between 200–350 cells/µl), and AIDS (CD4<200 cells/µl)) and injection drug use status (IDUs, non-IDUs), as well as prevention status (on MMT, not on MMT, on PrEP, not on PrEP) and HIV treatment status (on ART, not on ART). The model is illustrated in Figure S1 in Appendix S1; full details are provided in Appendix S1. Reflecting data from Ukraine, 1.6% of adults were IDUs, with 41.2% initial HIV prevalence among IDUs [14]–[19]. Initial HIV prevalence among non-IDUs was estimated to be 0.99% [9], [19]. The initial population distribution (IDU vs. non-IDU, distributed across HIV disease stages and ART status) was computed by applying the percentages shown in Table 1 to the 1,000,000 population (Table S3 in Appendix S1).

Individuals enter the population at age 15 into IDU and non-IDU compartments, and mature out of the population at age 49. We estimated that each year 1% of IDUs spontaneously quit injection drug use, and.03% of non-IDUs begin injection drug use [9]–[11], [20]. Individuals can die from non-AIDS-related causes (normal deaths, or drug-related deaths) or from AIDS.

We considered HIV transmission via injection equipment sharing (among IDUs) and sexual contacts (between any individuals). We calculated the risk of an uninfected IDU acquiring HIV via a risky injection based on the annual number of injections, percentage of injections involving shared equipment, the likelihood of sharing with an HIV-infected individual, and the probability of HIV transmission per risky injection (which depended on the HIV disease stage and ART status of the infected IDU, and PrEP status of the uninfected IDU).

We calculated the risk of sexual HIV transmission based on average number of annual sexual partnerships (using a higher value for IDUs than non-IDUs) [9], [21], [22], condom usage rate, condom effectiveness, and probability of transmission per risky partnership. Probability of transmission per risky partnership was calculated based on the HIV disease stage and ART status of the infected individual and, if relevant, the PrEP status of the uninfected individual.

Disease progression occurred at rates estimated from HIV natural history models [23]. We assumed that IDUs and non-IDUs with similar treatment status progressed at the same rates, with individuals on ART progressing more slowly than infected individuals not on ART.

### Antiretroviral Therapy

We assumed that individuals become eligible for ART when they develop late-stage HIV or AIDS (CD4<350 cells/µl) [24], [25]. Although very recent WHO guidelines [26] recommend initiation of treatment at CD4<500 cells/µl, we chose to model ART in a manner similar to its current usage. We accounted for the effects of ART on reducing disease progression and mortality [23], [27]–[29]. We assumed that ART reduces sexual infectivity by 96% [30], [31]. The extent to which ART reduces HIV infectivity from risky injection equipment sharing is unknown. Similar to previous analyses, we assumed a 50% reduction [9], [21]. We varied this value in sensitivity analysis. We estimated that ART would cost $450 annually for non-IDUs, with an additional $500 in counseling costs for IDUs not in MMT and $300 in counseling costs for IDUs in MMT [9], [21], [32]–[34].

### Methadone Maintenance Treatment

Based on available data regarding opiate substitution therapy in Ukraine [35] and elsewhere [36], and similar to a previous analysis of MMT in Ukraine [9], we estimated that IDUs in MMT reduced injection equipment sharing by 85% and had a higher likelihood of receiving ART in the absence of universal ART (25% for IDUs in MMT vs. 2% for IDUs not in MMT). We assumed that only 5% of individuals leaving MMT ceased injection drug use [9], [21]. We estimated that MMT (including counseling services) would cost $368 per client per year (comprising $168 for methadone and $200 for additional counseling and support services) [9], [21], [37].

### Pre-Exposure Prophylaxis

We assumed that PrEP would consist of a daily dose of TDF (300 mg)/FTC (200 mg) (Truvada). Consistent with the results of the recent clinical trial in Thailand, we assumed that PrEP would reduce the chance of acquiring HIV by 49% [7]. We assumed that this reduction would apply equally to sexual contacts and risky injections. Although behavioral disinhibition is a concern with PrEP use, there is no conclusive evidence regarding the effect of PrEP on risky sexual and needlesharing behavior [38]–[40]. In the base case, we assumed that IDUs receiving PrEP would have the same number of risky sexual and injection equipment sharing contacts as IDUs not receiving PrEP [39]–[41]; in sensitivity analysis, we considered both lower and higher levels of risk for IDUs on PrEP.

PrEP programs have not been implemented in Eastern Europe, so no cost data are available. In the absence of such data, we assumed that PrEP (with its associated counseling and monitoring) would cost approximately as much as ART for IDUs (with its associated counseling and monitoring). Because the Thai trial used directly observed therapy for much of the delivered PrEP [7], we assumed that, similar to ART, PrEP would have costs in addition to the drug cost; these include costs to monitor HIV status and PrEP side effects, to support medication adherence, etc. [2], [8]. Thus, in the base case we assumed that PrEP would cost $950 per person annually: this comprises a baseline ART cost of $450 (similar to the cost of ART for non-IDUs) plus $500 in additional IDU-related services for monitoring, adherence counseling, etc. [9], [21], [32]–[34]. We varied PrEP cost widely in sensitivity analysis.

### Model Calibration and Validation

We carried out extensive analyses to calibrate and validate the model, in order to verify its outputs and verify that the population dynamics accurately reflect population dynamics in Ukraine. We calibrated the model to match registered total HIV prevalence in Ukraine and other reported HIV epidemic data. Full details are provided elsewhere [9].

### Health Outcomes and Costs

For each prevention strategy, we calculated HIV prevalence, HIV infections averted, and QALYs gained. Quality multipliers (Table S2 in Appendix S1) were drawn from a previous study of HIV in Ukraine [9]. We took a societal perspective and considered all health care costs and savings, regardless of source or beneficiary [42]. In addition to the costs of the interventions, we included an annual health care cost of $311 for all individuals [43] and annual HIV care costs of $1,200 for all HIV-infected individuals [14]. We considered a 20-year time horizon, and discounted costs and QALYs to the present at 3%.

## Results

### Epidemic Impact

#### HIV prevalence

**Figure 1:**
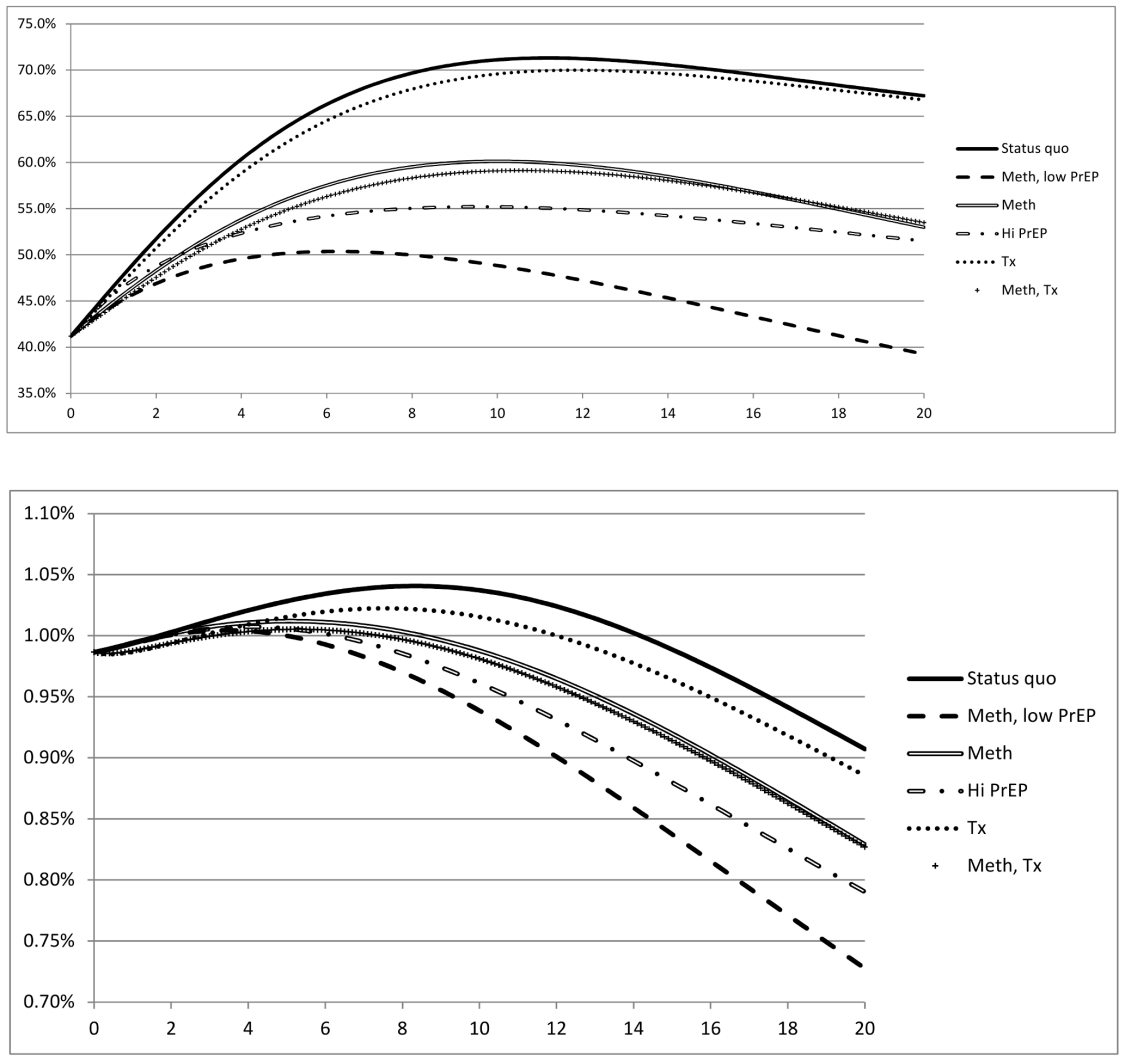
HIV prevalence over 20 years for alternative strategies. Prevalence is shown for the status quo and alternative strategies of scaling up antiretroviral therapy (ART) to 80% of all eligible individuals, methadone maintenance treatment (MMT) for 25% of injection drug users (IDUs), and introducing oral pre-exposure prophylaxis (PrEP) for 25% or 50% of uninfected IDUs.

Table 2 shows HIV prevalence after 20 years for all strategies. Figure 1 shows HIV prevalence over 20 years for the status quo and five of the strategies we considered. Without incremental interventions, HIV prevalence increased for approximately 10 years and then slowly decreased. Among IDUs (Figure 1a), HIV prevalence increased from an initial value of 41.2% to a maximum value of approximately 71% after 10 years, and then fell to 67.2% after 20 years. HIV prevalence among non-IDUs (Figure 1b) rose from 0.99% to a maximum value of approximately 1.04% after 10 years, and then fell to 0.91% after 20 years. The lowest HIV prevalence occurred when MMT and PrEP were combined: with MMT (for 25% of IDUs) and PrEP (for 50% of uninfected IDUs), HIV prevalence after 20 years was 33.9% in IDUs and 0.68% in non-IDUs. With the addition of ART to this portfolio, prevalence was very slightly higher because individuals on ART live longer than individuals not on ART.

**Table 2:** Base case results.*

		Single Interventions				Dual Interventions					All Interventions	
Outcome Measures	Status Quo	25% PrEP	50% PrEP	ART	MMT	MMT, 25% PrEP	MMT, 50% PrEP	MMT, ART	ART, 25% PrEP	ART, 50% PrEP	MMT, ART, 25% PrEP	MMT, ART, 50% PrEP
Number of individuals reached by each intervention over 20 yrs**												
PrEP	0	13,686	20,671	0	0	16,010	23,012	0	14,506	21,655	16,645	23,724
MMT	0	0	0	0	50,486	50,060	49,879	49,857	0	0	49,536	49,403
ART	10,620	10,166	9928	39,702	13,283	12,317	11,891	36,195	36,736	35,248	33,046	31,673
HIV prevalence after 20 yrs												
Among IDUs	67.2%	57.0%	51.5%	66.8%	53.0%	39.3%	33.9%	53.5%	55.8%	50.3%	39.5%	34.2%
Among non-IDUs	0.91%	0.83%	0.79%	0.89%	0.83%	0.73%	0.68%	0.83%	0.81%	0.77%	0.73%	0.69%
Overall	1.47%	1.34%	1.26%	1.50%	1.32%	1.11%	1.03%	1.36%	1.35%	1.27%	1.14%	1.05%
Infections averted												
Among IDUs	0	2355	3636	757	2982	6043	7384	3672	3308	775	6799	8115
Among non-IDUs	0	1196	1828	3179	1741	3087	3688	4492	4240	4539	5653	6152
Total	0	3552	5464	3935	4723	9130	11,072	8164	7548	9401	12,453	14,267
Incremental QALYS and cost***												
QALYs	–	29,294	44,963	96,323	75,458	111,032	127,018	163,772	122,422	135,821	194,720	208,102
Cost (US 1000s)	–	$40,405	$63,430	$94,864	$39,018	$106,937	$139,009	$127,788	$134,871	$157,026	$194,723	$225,521

* The table presents undiscounted HIV infections averted, and discounted QALYs and costs (discounted to the present at 3% annually). IDU = injection drug user; PrEP = oral pre-exposure prophylaxis for injection drug users; ART = antiretroviral therapy for 80% of eligible individuals; MMT = methadone maintenance treatment for 25% of IDUs; 25% PrEP = PrEP for 25% of uninfected IDUs; 50% PrEP = PrEP for 50% of uninfected IDUs.

** The numbers for similar strategies vary because of dynamics. For example, for the strategy “25% PrEP,” 13,686 IDUs receive PrEP, whereas for the strategy “ART, 25% PrEP,” 14,506 IDUs receive PrEP; this is because with scaled up ART, IDUs live longer, so 25% of IDUs on PrEP with ART scale up is greater than 25% of IDUs on PrEP without ART scale up.

*** Incremental to the status quo.

#### HIV infections averted

**Figure 2:**
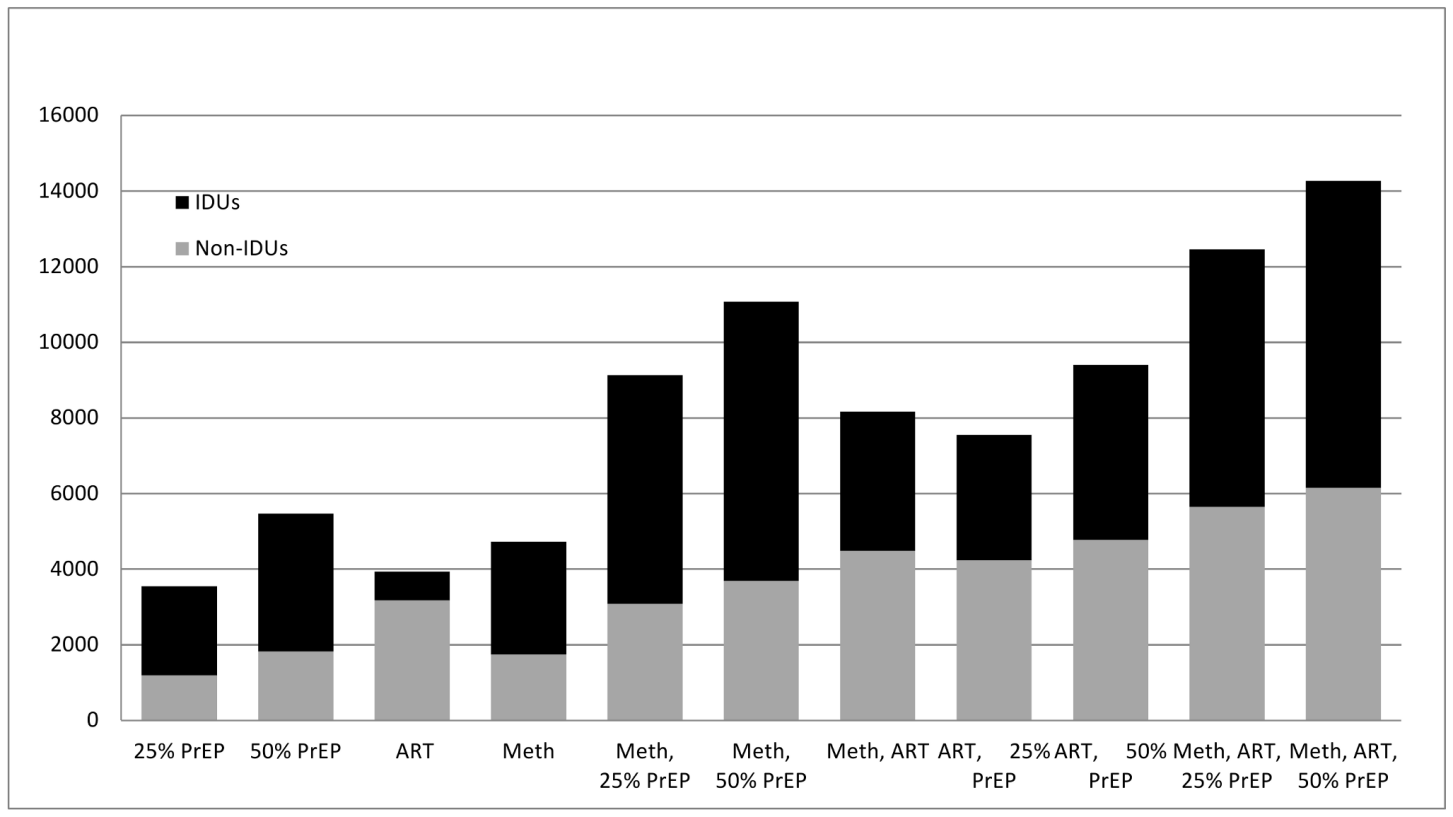
HIV infections averted over 20 years for alternative strategies. Infections averted are shown for alternative strategies of scaling up antiretroviral therapy (ART) to 80% of all eligible individuals, methadone maintenance treatment (MMT) to 25% of injection drug users (IDUs), and introducing oral pre-exposure prophylaxis (PrEP) for 25% or 50% of uninfected IDUs.

Table 2 and Figure 2 show the number of HIV infections averted for each strategy. When the interventions are implemented alone, PrEP at 50% coverage averts the greatest number of infections (5,464 infections over 20 years), followed by MMT (at 25% coverage; 4,723 infections averted), followed by universal ART coverage (3,935 infections averted), and then by PrEP at 25% coverage (3,552 infections averted). Figure 2 shows that the interventions targeted solely to IDUs–MMT and PrEP–averted significant numbers of HIV infections among non-IDUs. This is due to reduced sexual transmission of HIV from IDUs to non-IDU partners.

PrEP can enhance the prevention effects of MMT and ART: PrEP increased HIV infections averted by 93%–234% when added to MMT, and by 92%–239% when added to ART, depending on the level of PrEP coverage. PrEP and MMT are synergistic: when combined, PrEP and MMT averted more infections than either intervention alone. For example, when implemented alone, a program of 25% PrEP coverage averted 3,552 infections over 20 years, and an MMT program (with 25% coverage) averted 4,723 infections. When implemented together, these programs averted 9,130 infections–more than the sum of the benefits when the programs were implemented alone (8,275 = 3,552+4,723). MMT and PrEP work synergistically because MMT reduces injection equipment sharing significantly, and then PrEP further keeps the uninfected IDUs from acquiring the infection via sexual transmission.

When combined with ART, the benefits of PrEP are slightly less than additive: for example, the total number of infections averted when 25% PrEP coverage is combined with ART (7,548) is just slightly greater than the sum of the number of infections averted if PrEP and ART were to be implemented alone (7,487 = 3,552+3,935). When MMT and ART are combined, the benefits are also less than additive (8,164 infections averted when the interventions are implemented together; if implemented separately, 8,658 = 3,935+4,723). This is because there is significant overlap in the scope of these interventions – MMT and PrEP both reduce the chance of an uninfected IDU acquiring HIV infection and ART reduces the chance of an infected person transmitting infection.

As expected, combinations of the three interventions (rightmost columns of Table 2) reduced HIV incidence the most. Portfolios that include the three interventions averted slightly more infections than the sum of the interventions if implemented alone.

### Cost Effectiveness

**Figure 3:**
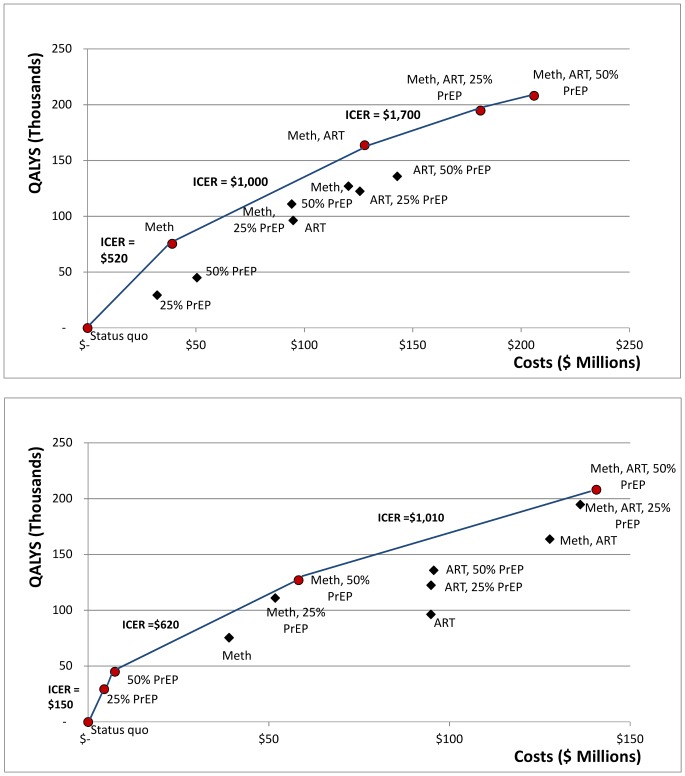
Cost effectiveness of alternative prevention and treatment strategies. Assumes annual PrEP cost of $950 (Figure 3a) and $450 (Figure 3b). PrEP = oral pre-exposure prophylaxis for injection drug users; ART = antiretroviral therapy for 80% of eligible individuals; MMT = methadone maintenance treatment for 25% of IDUs; 25% PrEP = PrEP for 25% of uninfected IDUs; 50% PrEP = PrEP for 50% of uninfected IDUs.

Figure 3a shows the estimated cost and effectiveness of each intervention strategy. Strategies of PrEP alone, PrEP combined with MMT alone, or PrEP combined with ART alone are dominated by strategies of MMT alone or MMT combined with ART. Among the undominated strategies, the most cost-effective strategy is MMT alone, with an incremental cost-effectiveness ratio (ICER) of approximately $520/QALY gained compared to the status quo. The next most cost-effective strategy is MMT combined with ART, which has an ICER of $1,000QALY gained compared to MMT alone. The World Health Organization suggests that interventions that cost less than a country’s GDP per capita are highly cost effective [32], [44]. Ukraine’s GDP per capita is approximately $7,400 [45], indicating that MMT alone, or MMT combined with ART are highly cost effective in this setting. Adding PrEP to a portfolio that includes MMT (at 25% coverage) and ART (at 80% coverage) costs approximately $1,700/QALY gained for 25% PrEP coverage and $2,300/QALY gained for an additional 25% PrEP coverage (thus 50% total coverage) – amounts that would be highly cost effective in this setting.

### Sensitivity Analysis

We performed one-way sensitivity analysis on all model parameters (ranges shown in Table 1). Infections averted were most sensitive to the following parameters: effectiveness of PrEP in reducing HIV acquisition and cost of PrEP (described below); and effectiveness of MMT in reducing risky injection behaviors, percentage of sexual contacts shared by IDUs with IDUs, and effectiveness of ART in reducing injection-related HIV transmission. Changes in the latter three parameters affected the effectiveness and cost effectiveness of the strategies we considered, but did not change the relative ranking of strategies (Appendix S1; Table S4 in Appendix S1).

#### PrEP effectiveness and adherence

The Bangkok Tenofovir Study estimated that daily oral PrEP for IDUs reduced HIV acquisition by 49%, with a 95% confidence interval of 10%–72% [7]. In that trial, many participants received directly observed therapy, which may have led to high adherence. If adherence in a different setting were lower, it is probable that the chance of infection acquisition would be higher [2]. If PrEP reduces HIV acquisition by only 10% (Table S4 in Appendix S1), then PrEP appears much less favorable than ART and MMT: for example, 509 infections averted by 25% PrEP coverage compared to 3,935 infections averted by ART (80% coverage) and 4,723 infections averted by MMT (at 25% coverage). Conversely, if PrEP reduces HIV acquisition by 72% (Table S4 in Appendix S1), then PrEP averts more infections than MMT or ART, even when only 25% of IDUs receive PrEP.

The base case assumed no change in risky sexual and injection equipment sharing behavior due to PrEP (and its associated counseling). If risky behavior increases by 25% for individuals on PrEP, then approximately 15% fewer infections are averted by strategies involving PrEP compared to the base case; and if risky behavior decreases by 25%, then approximately 15% more HIV infections are averted. Behavior change had a modest impact on the ICER: with 25% more risky behavior, the ICER of PrEP compared to the status quo increased from $1,100/QALY in the base case to $1,230; with 25% less risky behavior, the ICER decreased to $975.

#### Cost of PrEP

A key uncertainty affecting estimated cost effectiveness of the interventions is the cost of PrEP. Since PrEP has not been implemented in the region, it is not known how much it would cost. In the base case analysis, which assumed that PrEP costs $950 (similar to the cost of ART for IDUs in Ukraine), we found that PrEP is only cost effective (with an ICER of $1,700) when added to a portfolio that already includes 25% MMT coverage for IDUs and universal ART coverage. If the cost of PrEP is as high as $2,750 annually, it would still be highly cost effective to add PrEP to such a portfolio (ICER of $7,400). If the cost of PrEP is $650, then PrEP would be as cost effective as MMT ($520/QALY gained compared to the status quo), and if the cost of PrEP is $370 or less, PrEP would be cost saving.

Figure 3b shows the cost effectiveness of all strategies assuming a PrEP cost of $450. This corresponds to an estimated current minimum value of $350 per year for the drug regimen [46] and $100 per year for associated counseling and monitoring, likely the minimum cost achievable under current conditions. In this case, strategies involving PrEP for IDUs dominate strategies without PrEP. The most cost-effective strategy is to scale up PrEP (ICER $150), then to scale up MMT (ICER $620), and then to scale up ART (ICER $1,010).

#### PrEP cost and effectiveness

**Figure 4:**
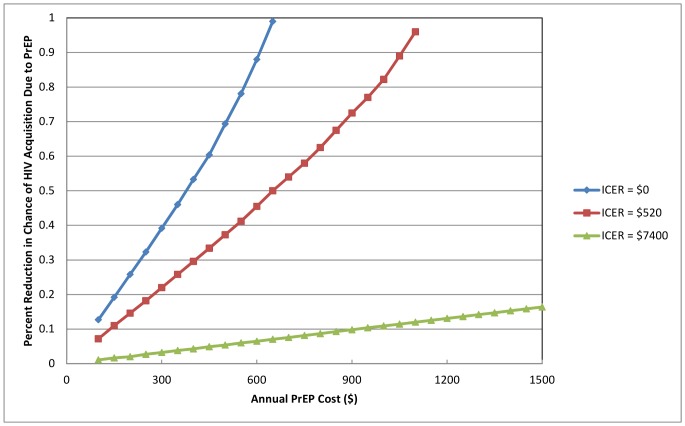
Breakeven effectiveness for PrEP as a function of its annual cost. Effectiveness is measured as percentage reduction in chance of HIV infection acquisition. Assumes 25% of uninfected IDUs are enrolled in PrEP, in comparison to the status quo (no scale up of MMT or ART). Bottom line (triangles) shows minimum effectiveness for which PrEP would be considered highly cost effective (ICER = $7,400). Middle line (diamonds) shows minimum effectiveness for which PrEP would be as cost effective as MMT (ICER = $520). Top line (squares) shows minimum effectiveness for which PrEP would be cost saving (ICER = $0). PrEP = oral pre-exposure prophylaxis for injection drug users; ART = antiretroviral therapy; MMT = methadone maintenance therapy; ICER = incremental cost-effectiveness ratio.

We performed a two-way sensitivity analysis on the cost of PrEP and its effectiveness in reducing the chance of HIV acquisition (Figure 4), assuming 25% of uninfected IDUs enrolled in PrEP, and comparing this to the status quo (no MMT or ART scale up). We calculated the breakeven reduction in HIV acquisition that would be required for PrEP to be cost saving (top line in Figure 4), to have the same ICER as MMT (middle line in Figure 4), and to be considered highly cost effective (bottom line in Figure 4). Thus, for example, if PrEP cost $300 per year, it would be cost saving if it were at least 39% effective in reducing the chance of HIV infection acquisition; it would be as cost effective as MMT if its effectiveness was 22%; and it would be considered highly cost effective if its effectiveness was at least 3.5%. If PrEP cost $600 per year, it could never be cost saving; it would be as cost effective as MMT if its effectiveness was 45%; and it would be considered highly cost effective if its effectiveness was at least 6.5%. If PrEP cost $1,200 per year, it would always be less cost effective than MMT but would be considered highly cost effective (cost per QALY gained less than Ukraine’s GDP per capita) if its effectiveness was at least 13%.

## Discussion

Our analysis of the effectiveness and cost effectiveness of ART, MMT, and PrEP for a mixed epidemic representative of Eastern Europe yielded several key findings. First, PrEP for 25% of uninfected IDUs averts fewer HIV infections than MMT (for 25% of IDUs) and ART (80% of all eligible individuals); but PrEP for 50% of IDUs averts more infections than MMT (for 25% of IDUs) and ART (80% of all eligible individuals). Second, PrEP significantly enhances the effect of MMT and ART programs: when added to large-scale ART and methadone programs, PrEP can prevent significant numbers of additional infections. Third, if the cost of PrEP is on a par with the cost of ART, then MMT and ART are more cost effective than PrEP for IDUs in an epidemic typical of Eastern Europe, but when PrEP for IDUs is added to a portfolio of prevention programs that already includes universal ART access and 25% coverage of MMT for IDUs, significant incremental benefits are generated at an affordable cost. If the cost of PrEP is about two-thirds the cost of ART, then introducing PrEP for IDUs without MMT or ART scale up is cost effective; and if the cost of PrEP is about half the cost of ART, then providing PrEP to IDUs would be cost saving.

We used 25% and 50% PrEP coverage as illustrative levels to represent moderate and relatively high coverage, respectively, and found that results are similar for both coverage levels. Thus, while PrEP coverage could be lower or higher in practice, depending on program reach, our policy conclusions would remain the same.

The Thai study authors estimated that the observed reductions in HIV incidence were due primarily to reductions in needlesharing risk, rather than reductions in sexual risk [7]. This is consistent with our analysis: IDUs face a higher risk of HIV acquisition from needlesharing (due to the high transmissibility of HIV via this route) than they do from sexual contacts. In the absence of information to the contrary, we assumed the same PrEP-induced reduction in infectivity for sexual and needlesharing contacts – but this still captures the greater reduction in overall risk of infection acquisition via needlesharing that the Thai study authors hypothesized.

A key question about effectiveness and cost effectiveness of PrEP in IDUs is the degree to which the reduction in HIV acquisition observed in the Bangkok Tenofovir Study [7] can be obtained in other settings and populations. Of note, that study used directly observed therapy for administration of PrEP for the majority of participants. The results of prior trials of PrEP suggest that adherence is a key determinant of effectiveness [2], [5], [6]. Whether directly observed therapy is necessary to achieve adherence sufficient to match the reductions in HIV transmission in the Bangkok Tenofovir Study is not known. To ensure that our estimates of cost effectiveness accounted for the potential need for interventions to improve adherence, our cost estimates included substantial additional costs beyond the cost of TDF/FTC. If such interventions were not needed to obtain adequate adherence, our analyses would underestimate the cost effectiveness of PrEP.

Our analysis also highlights that if PrEP can be provided relatively inexpensively, its use could reduce total expenditures on HIV care. Prevention of HIV transmission results in substantial downstream cost savings when assessed over time horizons sufficiently long to account for the savings. Our findings underscore the importance of efforts to lower the pharmaceutical cost of PrEP and of identifying approaches for improving adherence inexpensively outside the context of clinical trials.

PrEP is only one of several options for reducing HIV transmission in IDU populations. An important question for policymakers and clinicians is whether PrEP should be used, and if so, whether PrEP should be combined with other interventions. Our analysis addresses this question directly by considering the use of PrEP, MMT, and ART singly and in combinations. Our analyses enable us to assess whether interventions are synergistic, and under what conditions. Importantly, we find modest synergy between PrEP and MMT, suggesting that policymakers may wish to consider portfolios that include these two interventions if the cost of PrEP is low enough. Additionally, there may be cost synergies for individuals who receive both PrEP and MMT or for individuals who receive both ART and MMT.

The cost effectiveness of oral PrEP has been studied in other contexts, and a recent systematic review identified 13 studies of the cost effectiveness of PrEP [47]. Several studies have found that PrEP could be cost effective for MSM in both high- and middle-income countries if men at highest risk are reached [41], [48]–[50]. Studies of PrEP for heterosexuals in hyperendemic settings such as in sub-Saharan Africa have found that PrEP could be cost effective if targeted to the individuals with the highest sexual activity [51], but that PrEP is less cost effective than ART unless PrEP costs significantly less than ART [52]–[54]. In general, consistent with our findings, these studies suggest that cost-effective provision of PrEP depends on reaching high-risk individuals, ensuring adherence, and reducing drug costs.

Our analysis has several limitations. We did not distinguish between low-risk versus high-risk IDUs. If PrEP were targeted to IDUs most at risk for HIV infection, instead of IDUs at average risk as we have assumed, PrEP would be more cost effective than we have estimated. Additionally, we did not model network effects. Instead we assumed that, with the exception of preferential sexual mixing by IDUs (an estimated 45% of IDU sexual contacts are shared with other IDUs [21], [22], [55]–[57]), individuals mix homogeneously (thus, the probability of having a risky contact with another individual depended only on the relative size of the compartment that the other individual is in). The homogeneous mixing assumption may not hold in practice since IDUs are often involved in sexual and needle-sharing networks with other drug injectors, rather than mixing randomly. If MMT, PrEP and ART programs reach IDUs who are central in such networks, these programs likely will be more effective and cost effective than we have estimated; conversely, if the programs reach IDUs who are not central in such networks, they will likely be less effective and cost effective than we have estimated.

Previous studies have evaluated the potential cost effectiveness of oral PrEP among MSM [41], [48]–[50] and heterosexuals [51], [53], [54], [58], [59], either alone or in combination with other interventions. Our study is the first to examine the impact and cost effectiveness of oral PrEP among IDUs. Our analysis suggests that oral PrEP for IDUs can be part of an effective and cost-effective strategy to control HIV in regions where injection drug use is a significant driver of the epidemic. Where budgets are limited, focusing on MMT and ART access should be the priority, unless PrEP has low cost: at low cost (less than $650 per year in Ukraine), oral PrEP alone could be highly cost effective.

## Supporting Information

Appendix S1 **Supporting figures and tables.** Figure S1 Schematic of model. Table S1 Summary of notation for parameters and variables. Table S2 Parameter values, ranges and sources. Table S3 Initial population distribution for the model. Table S4 HIV infections averted: results of one-way sensitivity analyses.
(DOCX)
