# Supplementary material for: Effectiveness and Cost Effectiveness of Oral Pre-Exposure Prophylaxis in a Portfolio of Prevention Programs for Injection Drug Users in Mixed HIV Epidemics
Source: PLoS One. 2014 Jan 28;9(1):e86584. doi: 10.1371/journal.pone.0086584 (PMC3904940; doi:10.1371/journal.pone.0086584)
Supplement: Appendix S1 — Supporting figures and tables. Figure S1 Schematic of model. Table S1 Summary of notation for parameters and variables. Table S2 Parameter values, ranges and sources. Table S3 Initial population distribution for the model. Table S4 HIV infections averted: results of one-way sensitivity analyses. (DOCX) [file pone.0086584.s001.docx]

### Effectiveness and Cost Effectiveness of Oral Pre-exposure Prophylaxis in a Portfolio of Prevention Programs for Injection Drug Users in Mixed HIV Epidemics

S.S. Alistar, D.K. Owens, M.L. Brandeau

### APPENDIX S1

**I. Epidemic Model**

**Model overview**

The model is an extension of a previously published model used to assess scale up of antiretroviral therapy (ART) and methadone maintenance treatment (MMT) in Ukraine [[1](#_ENREF_1)]. We extended the previous model to allow for HIV-uninfected injection drug users (IDUs) to receive oral pre-exposure prophylaxis (PrEP). The model is illustrated schematically in Figure S1. Model notation is presented in Table S1. The model is a dynamic compartmental model, given by a system of non-linear differential equations that describe the change in number of individuals in each compartment over time. We let *X_i_*(*t*) denote the number of individuals in compartment *i* (*i* = 1, …, 20) at time *t* (see Figure S1). Transitions between compartments, and into or out of the population, occur at rates defined by demographic parameters, disease progression parameters, and resource availability (ART, MMT, PrEP). All rates were estimated on a yearly basis. Parameter values are shown in Table S2, and the initial distribution of the population among the compartments at time 0 (*X*_1_(0), …, *X*_20_(0)) is shown in Table S3.

In the model implementation we used a 20-year time horizon and discretized the differential equations into difference equations using time steps of 1/10 year; thus, in the discretized implementation, *t* = 1, …, 200. We implemented the model in Microsoft Excel.

**Model equations**

The differential equations describing the model dynamics are as follows.

Uninfected IDUs not in MMT and not receiving PrEP:

$$\frac{{dX}_{1}\left( t \right)}{dt}={}_{1}+{}_{2,1}\left( t \right)X_{2}\left( t \right)+{}_{8,1}\left( t \right)X_{8}\left( t \right)+{}_{15,1}\left( t \right)X_{15}\left( t \right)-X_{1}\left( t \right)\left[ {}_{1,2}\left( t \right)+{}_{1,8}\left( t \right)+{}_{1,15}\left( t \right)+_{1}+{}_{1}+\sum_{j=1}^{20} \left( {}_{1j}^{I}+{}_{1j}^{S} \right)X_{j}\left( t \right) \right]$$

Uninfected IDUs not in MMT, but receiving PrEP:

$$\frac{{dX}_{2}\left( t \right)}{dt}={{}_{1,2}\left( t \right)X_{1}\left( t \right)+{}_{9,2}\left( t \right)X_{9}\left( t \right)-X}_{2}\left( t \right)\left[ {}_{2,1}\left( t \right)+{}_{2,9}\left( t \right)+_{2}+{}_{2}+\sum_{j=1}^{20} \left( {}_{2j}^{I}+{}_{2j}^{S} \right)X_{j}\left( t \right) \right]$$

IDUs with early HIV not in MMT:

$$\frac{{dX}_{3}\left( t \right)}{dt}={}_{10,3}\left( t \right)X_{10}\left( t \right)+{}_{16,3}\left( t \right)X_{16}\left( t \right)+X_{1}\left( t \right)\left[ \sum_{j=1}^{20} \left( {}_{1j}^{I}+{}_{1j}^{S} \right)X_{j}\left( t \right) \right]+X_{2}\left( t \right)\left[ \sum_{j=1}^{20} \left( {}_{2j}^{I}+{}_{2j}^{S} \right)X_{j}\left( t \right) \right]{- X}_{3}\left( t \right)\left[ {}_{3,10}\left( t \right)+{}_{3,16}\left( t \right) {+ {}_{3,4}+}_{3}+{}_{3} \right]$$

IDUs with late HIV not in MMT and not receiving ART:

$$\frac{{dX}_{4}\left( t \right)}{dt}={{}_{3,4}X_{3}\left( t \right)+}_{6,4}\left( t \right)X_{6}\left( t \right)+{}_{11,4}\left( t \right)X_{11}\left( t \right)+{}_{17,4}\left( t \right)X_{17}\left( t \right){- X}_{4}\left( t \right)\left[ {}_{4,6}\left( t \right)+{}_{4,11}\left( t \right) + {}_{4,17}\left( t \right)+ {}_{4,5}+ {}_{4}+{}_{4}+ {}_{4} \right]$$

IDUs with AIDS not in MMT and not receiving ART:

$$\frac{{dX}_{5}\left( t \right)}{dt}={{}_{4,5}X_{4}\left( t \right)+}_{7,5}\left( t \right)X_{7}\left( t \right)+{}_{12,5}\left( t \right)X_{12}\left( t \right)+{}_{18,5}\left( t \right)X_{18}\left( t \right){- X}_{5}\left( t \right)\left[ {}_{5,7}\left( t \right)+{}_{5,12}\left( t \right) + {}_{5,18}\left( t \right)+ {}_{5}+{}_{5}+ {}_{5} \right]$$

IDUs with late HIV, not in MMT but on ART:

$$\frac{{dX}_{6}\left( t \right)}{dt}={}_{4,6}\left( t \right)X_{4}\left( t \right)+{}_{13,6}\left( t \right)X_{13}\left( t \right)+{}_{19,6}\left( t \right)X_{19}\left( t \right){- X}_{6}\left( t \right)\left[ {}_{6,4}\left( t \right)+{}_{6,13}\left( t \right) + {}_{6,19}\left( t \right)+ {}_{6,7}+ {}_{6}+{}_{6}+ {}_{6} \right]$$

IDUs with AIDS not in MMT but on ART:

$$\frac{{dX}_{7}\left( t \right)}{dt}={{}_{6,7}X_{6}\left( t \right)+}_{5,7}\left( t \right)X_{5}\left( t \right)+{}_{14,7}\left( t \right)X_{14}\left( t \right)+{}_{20,7}\left( t \right)X_{20}\left( t \right){- X}_{7}\left( t \right)\left[ {}_{7,5}\left( t \right)+{}_{7,14}\left( t \right) + {}_{7,20}\left( t \right)+ {}_{7}+{}_{7}+ {}_{7} \right]$$

Uninfected IDUs in MMT but not receiving PrEP:

$$\frac{{dX}_{8}\left( t \right)}{dt}={}_{1,8}\left( t \right)X_{1}\left( t \right)+{}_{9,8}\left( t \right)X_{9}\left( t \right)+{}_{15,8}\left( t \right)X_{15}\left( t \right)-X_{8}\left( t \right)\left[ {}_{8,1}\left( t \right)+{}_{8,9}\left( t \right)+{}_{8,15}\left( t \right) +_{8}+{}_{8}+\sum_{j=1}^{20} \left( {}_{8j}^{I}+{}_{8j}^{S} \right)X_{j}\left( t \right) \right]$$

Uninfected IDUs in MMT and receiving PrEP:

$$\frac{{dX}_{9}\left( t \right)}{dt}={{}_{2,9}\left( t \right)X_{2}\left( t \right)+{}_{8,9}\left( t \right)X_{8}\left( t \right)-X}_{9}\left( t \right)\left[ {}_{9,2}\left( t \right)+{}_{9,8}\left( t \right)+_{9}+{}_{9}+\sum_{j=1}^{20} \left( {}_{9j}^{I}+{}_{9j}^{S} \right)X_{j}\left( t \right) \right]$$

IDUs with early HIV in MMT:

$$\frac{{dX}_{10}\left( t \right)}{dt}={}_{3,10}\left( t \right)X_{3}\left( t \right)+X_{8}\left( t \right)\left[ \sum_{j=1}^{20} \left( {}_{8j}^{I}+{}_{8j}^{S} \right)X_{j}\left( t \right) \right]+X_{9}\left( t \right)\left[ \sum_{j=1}^{20} \left( {}_{9j}^{I}+{}_{9j}^{S} \right)X_{j}\left( t \right) \right]{- X}_{10}\left( t \right)\left[ {}_{10,3}\left( t \right){+ {}_{10,16}\left( t \right)+{}_{10,11}+}_{10}+{}_{10} \right]$$

IDUs with late HIV in MMT, not receiving ART:

$$\frac{{dX}_{11}\left( t \right)}{dt}={{}_{10,11}X_{10}\left( t \right)+}_{4,11}\left( t \right)X_{4}\left( t \right)+{}_{13,11}\left( t \right)X_{13}\left( t \right){- X}_{11}\left( t \right)\left[ {}_{11,4}\left( t \right)+{}_{11,13}\left( t \right) + {}_{11,17}\left( t \right)+ {}_{11,12}+ {}_{11}+{}_{11}+ {}_{11} \right]$$

IDUs with AIDS in MMT, not receiving ART:

$$\frac{{dX}_{12}\left( t \right)}{dt}={{}_{11,12}X_{11}\left( t \right)+}_{5,12}\left( t \right)X_{5}\left( t \right)+{}_{14,12}\left( t \right)X_{14}\left( t \right){- X}_{12}\left( t \right)\left[ {}_{12,5}\left( t \right)+{}_{12,14}\left( t \right) + {}_{12,18}\left( t \right)+ {}_{12}+{}_{12}+ {}_{12} \right]$$

IDUs with late HIV in MMT and on ART:

$$\frac{{dX}_{13}\left( t \right)}{dt}={}_{6,13}\left( t \right)X_{6}\left( t \right)+{}_{11,13}\left( t \right)X_{11}\left( t \right){- X}_{13}\left( t \right)\left[ {}_{13,6}\left( t \right)+{}_{13,11}\left( t \right) + {}_{13,19}\left( t \right)+ {}_{13,14}+ {}_{13}+{}_{13}+ {}_{13} \right]$$

IDUs with AIDS in MMT and on ART:

$$\frac{{dX}_{14}\left( t \right)}{dt}={{}_{13,14}X_{13}\left( t \right)+}_{7,14}\left( t \right)X_{7}\left( t \right)+{}_{12,14}\left( t \right)X_{12}\left( t \right){- X}_{14}\left( t \right)\left[ {}_{14,7}\left( t \right)+{}_{14,12}\left( t \right) + {}_{14,20}\left( t \right)+ {}_{14}+{}_{14}+ {}_{14} \right]$$

Uninfected non-IDUs:

$$\frac{{dX}_{15}\left( t \right)}{dt}={}_{15}+{}_{1,15}\left( t \right)X_{1}\left( t \right)+{}_{8,15}\left( t \right)X_{8}\left( t \right)-X_{15}\left( t \right)\left[ {}_{15,1}\left( t \right)+_{15}+{}_{15}+\sum_{j=1}^{20} \left( {}_{15,j}^{S} \right)X_{j}\left( t \right) \right]$$

Non-IDUs with early HIV:

$$\frac{{dX}_{16}\left( t \right)}{dt}={}_{3,16}\left( t \right)X_{3}\left( t \right)+{}_{10,16}\left( t \right)X_{10}\left( t \right)+X_{15}\left( t \right)\left[ \sum_{j=1}^{20} \left( {}_{15,j}^{S} \right)X_{j}\left( t \right) \right]{- X}_{16}\left( t \right)\left[ {}_{16,3}\left( t \right) {+ {}_{16,17}+}_{16}+{}_{16} \right]$$

Non-IDUs with late HIV not receiving ART:

$$\frac{{dX}_{17}\left( t \right)}{dt}={{}_{16,17}X_{16}\left( t \right)+}_{4,17}\left( t \right)X_{4}\left( t \right) +{}_{11,17}\left( t \right)X_{11}\left( t \right) + {}_{19,17}X_{19}\left( t \right){- X}_{17}\left( t \right)\left[ {}_{17,4}\left( t \right)+ {}_{17,19}\left( t \right)+ {}_{17,18}+ {}_{17}+{}_{17}+ {}_{17} \right]$$

Non-IDUs with AIDS not receiving ART:

$$\frac{{dX}_{18}\left( t \right)}{dt}={{}_{17,18}X_{17}\left( t \right)+}_{5,18}\left( t \right)X_{5}\left( t \right)+{}_{12,18}\left( t \right)X_{12}\left( t \right){+{}_{20,18}\left( t \right)X_{20}\left( t \right)- X}_{18}\left( t \right)\left[ {}_{18,5}\left( t \right)+{}_{18,20}\left( t \right) + {}_{18}+{}_{18}+ {}_{18} \right]$$

Non-IDUs with late HIV on ART:

$$\frac{{dX}_{19}\left( t \right)}{dt}={}_{6,19}\left( t \right)X_{6}\left( t \right)+{}_{13,19}\left( t \right)X_{13}\left( t \right) +{}_{17,19}\left( t \right)X_{17}\left( t \right) {- X}_{19}\left( t \right)\left[ {}_{19,6}\left( t \right)+ {}_{19,17}\left( t \right)+ {}_{19,20}+ {}_{19}+{}_{19}+ {}_{19} \right]$$

Non-IDUs with AIDS on ART:

$$\frac{{dX}_{20}\left( t \right)}{dt}={}_{19,20}X_{19}\left( t \right)+{}_{7,20}\left( t \right)X_{7}\left( t \right)+{}_{14,20}\left( t \right)X_{14}\left( t \right) +{}_{18,20}\left( t \right)X_{18}\left( t \right) {- X}_{20}\left( t \right)\left[ {}_{20,7}\left( t \right)+ {}_{20,18}\left( t \right)+ {}_{20}+{}_{20}+ {}_{20} \right]$$

**Description of model dynamics**

Individuals enter the model at age 15 (14-year-olds turning 15) into the uninfected non-IDU compartment (at rate *ρ*_15_) and the uninfected IDU compartment (at rate *ρ*_1_). We set the ratio *ρ*_1_/(*ρ*_1_+* *_15_)such that the percentage of IDUs in the population remained constant. We assumed that all new entrants to the population are uninfected with HIV. Individuals may leave the population if they die of non-AIDS related causes at rates *δ_i_* from any compartment *i*, or from advanced HIV/AIDS at rate *α_i_* from compartments in which individuals have late HIV or AIDS. At age 49, individuals mature out of the population at rate *μ_i_* from each compartment *i*.

Once infected, individuals progress through the HIV disease stages (early HIV to late HIV, late HIV to AIDS) at rates *θ_i,j_* between compartments *i* and *j*. These rates are computed as the reciprocal of the typical time spent in each stage, calculated from a model of the natural history of HIV [[2](#_ENREF_2)]. ART lowers disease progression rates *θ_i,j_* by increasing the time spent in each disease stage, and also lowering the AIDS death rate *α_I_* [[3-5](#_ENREF_3)]. Thus, the AIDS death rate for individuals in the untreated AIDS compartments is higher than the AIDS death rate for individuals in treated compartments*.* We assumed that drug usage status and methadone treatment do not affect the evolution of the disease. Individuals become more infectious as they progress in the disease, in the absence of ART.

In computing HIV transmission rates we took an approach similar to Alistar et al. [[1](#_ENREF_1)] and Long et al. [[3](#_ENREF_3)], but modified the calculations to allow for potential transmission reductions due to PrEP. Non-IDUs can acquire HIV only through risky sexual contacts with infected individuals. Condom usage (40% for IDUs not in MMT, 45% for non-IDUs and for IDUs in MMT [[1](#_ENREF_1), [3](#_ENREF_3), [6-8](#_ENREF_6)]) reduces the riskiness of sexual contacts, but is only 90% effective [[3](#_ENREF_3), [9-11](#_ENREF_9)]. IDUs can acquire HIV infection either through risky sexual contacts or by sharing injection equipment with an infected individual (an estimated 25% of injections are with shared equipment [[1](#_ENREF_1), [3](#_ENREF_3), [6-8](#_ENREF_6), [12](#_ENREF_12)]). The number of individuals in a given uninfected compartmentwho acquire HIV at any time *t* is a function of the sufficient contact rates of individuals in those compartments with infected individuals. The sufficient contact rates for risky sexual or equipment sharing encounters between individuals are computed as the product of the number of risky contacts of each kind and the probability that the contact is with an individual in an infected compartment.

The sufficient injection equipment sharing rate (i.e., the sharing rate sufficient to transmit HIV infection) for contacts between an uninfected IDU in compartment *i* and an infected IDU in compartment *j* is computed as:

$${}_{i,j}^{I}=I_{i}S_{i}\left[ \frac{{X_{j}I}_{j}S_{j}}{\sum_{k=1}^{14} {X_{k}I}_{k}S_{k}} \right]\left( {}_{ij}^{I}{}_{j}^{I}{}_{i}^{I} \right)$$

The sufficient needle sharing contact rate (${}_{i,j}^{I}$) is obtained by multiplying the number of shared injections of individuals in compartment *i* (*I_i_S_i_*) with the probability that the injection is shared with an individual from infected compartment *j* (the bracketed term in the above expression) and the probability that the infection is transmitted during a risky shared injection with a person in compartment *j* (${}_{i,j}^{I}{}_{i}$). The quantity (${}_{ij}^{I}{}_{j}^{I}{}_{i}^{I}$) is the chance of infection acquisition by individuals not on PrEP from individuals not on ART (${}_{i,j}^{I}$) multiplied by a factor denoting the reduction in the chance of infection transmission via risky injections for individuals on ART (${}_{j}^{I}$, a quantity that is less than 1 only for compartments with individuals on ART) and a factor denoting the reduction in the chance of infection acquisition via risky shared injections for individuals on PrEP (${}_{i}^{I}$, a quantity that is less than 1 only for compartments *i* = 2, 9).

We considered two types of risky sexual contacts, depending on whether a condom was used and was ineffective (low-risk contact) or was not used at all (high-risk contact). The sufficient sexual contact rates ${}_{i,j}^{S}$, which is calculated as the sum of sufficient sexual contact from low-risk partnerships ${}_{i,j}^{S,LR}$ and from high-risk partnerships ${}_{i,j}^{S,HR}$:

$${}_{i,j}^{S}={}_{i,j}^{S,LR}+{}_{i,j}^{S,HR}$$

We calculate ${}_{i,j}^{S,LR}$ and ${}_{i,j}^{S,LR}$as follows:

$${}_{i,j}^{S,LR}=P_{i}U_{i}\left( 1-ce \right)\left( {}_{ij}^{S}{}_{j}^{S}{}_{i}^{S} \right)F_{ij}$$

$${}_{i,j}^{S,LR}=P_{i}\left( 1-U_{i} \right)\left( {}_{ij}^{S}{}_{j}^{S}{}_{i}^{S} \right)F_{ij}$$

The above rates are obtained by multiplying: the number of yearly sexual partners $\left( P_{i} \right)$ with the probability of using a condom $\left( U_{i} \right)$ which fails $\left( 1-ce \right)$ (low risk) or the probability of not using a condom $\left( 1-U_{i} \right)$ (high risk); the probability of acquiring HIV per partnership (${}_{i,j}^{S}{}_{i})$; and the probability that a sexual partner is from compartment *j* ($F_{ij}$). Similar to the calculation of transmission probability via injection equipment sharing, the quantity $\left( {}_{ij}^{S}{}_{j}^{S}{}_{i}^{S} \right)$ is the chance of infection acquisition via risky sexual partnership by individuals not on PrEP from individuals not on ART $\left( {}_{ij}^{S} \right)$ multiplied by a factor denoting the reduction in the chance of infection transmission via risky sexual partnerships for individuals on ART (${}_{j}^{S}$, a quantity that is less than 1 only for compartments with individuals on ART) and a factor denoting the reduction in the chance of infection acquisition via risky sexual partnerships for individuals on PrEP (${}_{i}^{S}$, a quantity that is less than 1 only for compartments *i* = 2, 9).

The probabilities $F_{ij}$ are computed as follows:

$F_{ij}=\left( \text{Aff} \right)\left[ \frac{{X_{j}I}_{j}S_{j}}{\sum_{k=1}^{14} {X_{j}I}_{j}S_{j}} \right]$, *i* = 1, …, 14, *j* = 1, …, 14

$F_{ij}=\left( \text{1-}\text{Aff} \right)\left[ \frac{{X_{j}I}_{j}S_{j}}{\sum_{k=15}^{20} {X_{j}I}_{j}S_{j}} \right]$, *i* = 1, …, 14, *j* = 15, …, 20

$F_{ij}=\left( \text{1-}\text{Aff} \right)\left[ \frac{{X_{j}I}_{j}S_{j}}{\sum_{k=1}^{14} {X_{j}I}_{j}S_{j}} \right]$, *i* = 15, …, 20, *j* = 1, …, 14

$F_{ij}=1-\left\{ \left( \text{1}\text{-}\text{Aff} \right)\left[ \frac{{X_{j}I}_{j}S_{j}}{\sum_{k=15}^{20} {X_{j}I}_{j}S_{j}} \right] \right\}$, *i* = 15, …, 20, *j* = 15, …, 20

The above equations reflect the preferential mixing in sexual contacts of IDUs with other IDUs, expressed by the fraction *Aff*. In the base case we set this value to 0.45 [[3](#_ENREF_3), [6-8](#_ENREF_6), [12](#_ENREF_12)]. For any given compartment *i* the above probabilities sum up to 1 over all *j*.

**ART access**

ART is available to individuals whose disease has advanced to late HIV or AIDS (CD4 cell count below 350 cells/µl). We assumed different rates of baseline access to ART for IDUs not on MMT (2% of eligible individuals), IDUs on MMT (25% of eligible individuals), and non-IDUs (22% of eligible individuals); and different rates of quitting ART for IDUs not on MMT (65% annually), IDUs on MMT (40% annually), and non-IDUs (12.5% annually). When treatment is started, the individual transitions to the corresponding “treatment” compartment (e.g., an individual from compartment 4 transitions to compartment 6); and when exiting treatment, individuals transition to the corresponding “untreated” compartment (e.g., and individual from compartment 6 transitions to compartment 4). We assumed a fraction of eligible individuals are recruited into ART at each time step, depending on the scenario: either the baseline rates or 80% for universal access.

**Methadone maintenance treatment (MMT) access**

We assumed that a fixed fraction of IDUs receive MMT in any time period, depending on the scenario (either 0% under the status quo or 25% under MMT scale up), and that these fractions are the same within each disease state. In each period, some individuals quit MMT and return to injection drug use and some individuals successfully “graduate” from MMT. These are replaced in the next period by IDUs who were not on MMT in the previous period, such that an approximately constant fraction of IDUs receives MMT. Thus, under the status quo, *X*_8_(*t*), *X*_9_(*t*), …, *X*_14_(*t*) = 0; and under MMT scale up, *X*_8_(*t*) = 0.25*X*_1_(*t*), *X*_9_(*t*) = 0.25*X*_2_(*t*), *X*_10_(*t*) = 0.25*X*_3_(*t*), (*X*_11_(*t*) + *X*_13_(*t*)) = 0.25(*X*_4_(*t*) + *X*_6_(*t*)), and (*X*_12_(*t*) + *X*_14_(*t*)) = 0.25(*X*_5_(*t*) + *X*_7_(*t*)).

**Pre-exposure prophylaxis (PrEP) access**

We assumed that a fixed fraction of HIV-uninfected IDUs are recruited into PrEP in each time period (0%, 25%, or 50% of such IDUs, depending on the scenario), and that the same proportion of IDUs not in MMT receive PrEP as IDUs in MMT.

**Health outcomes and costs**

We computed the total costs in US dollars and benefits measured in QALYs over 20 years, discounted to the present using a 3% annual interest rate, for the status quo and all the considered strategies. To do so, we calculated total QALYs and costs incurred in each time period using the quality multipliers and cost values shown in Table S2. We also included future lifetime discounted costs and QALYs for all individuals alive in the population at the end of the time horizon.

We calculated incremental cost-effectiveness ratios (ICERs) by dividing incremental costs by incremental QALYs gained:

*ICER*_strategy A_=**

We calculated HIV prevalence at time *t* for the total population

*HIV Prevalence* _total_ *=* ,

the IDU population

*HIV Prevalence* _IDU_ *=*  ,

and the non-IDU population

*HIV Prevalence* _non-IDU_ *=* .

To compute the number of HIV infections averted, we computed for each strategy and the status quo the number of new infections occurring at each time *t* and over the entire time horizon. The number of infections averted by a strategy is the difference between the total number of new infections under the strategy, and the total number of new infections in the status quo.

**II. Supplemental Sensitivity Analyses**

Results of supplemental sensitivity analyses are shown in Table S4. These are parameters to which the estimated effectiveness (HIV infections averted) of different strategies was most sensitive. The sensitivity analysis on PrEP effectiveness (first rows of Table S4) is discussed in the main manuscript.

**Effectiveness of MMT**. The base case assumed that IDUs in MMT reduced risky injection equipment sharing by 85% [[10](#_ENREF_10), [11](#_ENREF_11), [13-15](#_ENREF_13)]. If the reduction is only 60%, MMT averts fewer infections than PrEP (even at 25% coverage) and ART (at 80% coverage). If the reduction is 99%, then MMT (for 25% of IDUs) averts more infections than PrEP for 50% of uninfected IDUs.

**Sexual Mixing Patterns**. We estimated that 45% of IDU sexual contacts are with other IDUs [[1](#_ENREF_1), [3](#_ENREF_3), [6-8](#_ENREF_6)]. If this value is lower (and thus IDUs have relatively more sexual contacts with non-IDUs), then all strategies avert more infections than in the base case. This is because there is more mixing between IDUs and non-IDUs—and thus relatively more potential transmission from IDUs to the general population. Conversely, if the value is higher (and IDUs have relatively fewer sexual contacts with non-IDUs), then all strategies avert fewer infections than in the base case. However, changing this value had little effect on the relative ranking of strategies in terms of HIV infections averted.

**Effect of ART on HIV Transmission**. No clinical trials have examined the effectiveness of ART in reducing HIV transmission via risky needlesharing contacts. In the base case we estimated that ART would reduce the risk of HIV transmission via risky injection equipment sharing by 50%. If this value is only 10%, then ART is less effective at reducing HIV incidence compared to PrEP and MMT than in the base case, whereas if this value is 90%, then ART is relatively more effective compared PrEP and MMT than in the base case. In both cases, however, the relative ranking of strategies in terms of HIV infections averted remained unchanged.

**Figure S1.** Schematic of model.

IDU = injection drug user; MMT = methadone maintenance treatment; ART = antiretroviral therapy; PrEP = oral pre-exposure prophylaxis. For simplicity, some allowable transitions (some vertical arrows between IDUs in MMT, IDUs not in MMT, and non-IDUs) are not shown in the figure.

**Table S1**. **Summary of notation for parameters and variables.**

**Indices**

*i*, *j* Index for compartments (*i*, *j* = 1,… 20)

*t* Time index (*t*≥0)

**Population compartments**

*X_i_*(*t*) Number of individuals in compartment *i* at time *t* (*i* = 1, …, 20)

**Parameters**

*ρ_i_* Rate of entry to compartment *i*, representing maturation into the population (*ρ_i_* = 0 for *i* ≠ 1, 15)

*μ_i_* Rate of maturation out of compartment *i*

*δ_i_* Rate of non-AIDS death for individuals in compartment *i*

*α_i_*AIDS death rate from compartment *i*(*α_i_* = 0 for uninfected and early HIV compartments)

*θ_i,j_*Rate of HIV disease progression from compartment *i* (with early or late HIV) to compartment *j* (with late HIV or AIDS)

*ϕ_i,j_*(*t*)Rate of transition between compartments *i* and *j* having different drug use status (IDU vs. non-IDU) or intervention status (PrEP vs. no PrEP, MMT vs. no MMT, ART vs. no ART), at time *t*

**Injection drug use parameters**

${}_{i,j}^{I}$Rate of injection equipment sharing contact between an uninfected individual in compartment *i* and an infected individual in compartment *j* that is sufficient to transmit HIV infection

*I_i_*Number of opiate injections per year by individuals in compartment *i*

*S_i_* Percentage of injections by individuals in compartment *i* that are shared

${}_{i,j}^{I}$ Probability of transmitting HIV via risky injection contact to an individual in compartment *i*from an individual in compartment *j*

${}_{j}^{I}$Multiplier denoting the percentage reduction due to ART in chance of HIV transmission via risky injections from an individual in compartment *j* (${}_{j}^{I}$ = 1 for non-ART compartments, denoting no reduction)

${}_{i}^{I}$Multiplier denoting the percentage reduction due to PrEP in chance of HIV acquisition via risky injections for an individual in compartment *i* (${}_{i}^{I}$ = 1 for non-PrEP compartments, denoting no reduction)

**Sexual behavior parameters**

${}_{i,j}^{S}$ Rate of risky sexual contact between an uninfected individual in compartment *i* and an infected individual in compartment *j* that is sufficient to transmit HIV infection

${}_{i,j}^{S, LR}$ Rate of risky sexual contact in a low-risk partnership (condom was used and failed) between an uninfected individual in compartment *i* and an infected individual in compartment *j*

${}_{i,j}^{S, HR}$ Rate of risky sexual contact in a high-risk partnership (condom was not used) between an uninfected individual in compartment *i* and an infected individual in compartment *j*

*P_i_* Number of sexual partners for an individual in compartment *i*

*U_i_* Condom usage rate for an individual in compartment *i*

*ce* Condom effectiveness

${}_{i,j}^{I}$ Probability of transmitting HIV in a risky sexual partnership to an individual in compartment *i*from an individual in compartment *j*

${}_{j}^{S}$Multiplier denoting the percentage reduction due to ART in chance of HIV transmission via risky sexual contacts from an individual in compartment *j* (${}_{j}^{S}$ = 1 for non-ART compartments, denoting no reduction)

${}_{i}^{S}$Multiplier denoting the percentage reduction due to PrEP in chance of HIV acquisition via risky sexual contacts for an individual in compartment *i* (${}_{i}^{S}$ = 1 for non-PrEP compartments, denoting no reduction)

*F_ij_* Probability an individual in compartment *i* has a sexual partnership with an individual in compartment *j*

*Aff* Percentage of sexual partners of IDUs who are also IDUs

**Table S2**. **Parameter values, ranges and sources.**

| **Parameter** | **Value** | **Range** | **Source** |
| --- | --- | --- | --- |
| **Population** |  |  |  |
| Initial population (age 15-49) | 1,000,000 |  |  |
| Proportion IDUs | 1.60% | 1.34%–1.75% | [[16](#_ENREF_16)] |
| **Prevalence** |  |  |  |
| Initial HIV prevalence IDUs | 41.20% | 17.3%–70.0% | [[16-21](#_ENREF_16)] |
| Initial HIV prevalence non-IDUs | 0.99% | 0.73%–1.16% | Calculated |
| **Initial disease stages distribution** |  |  |  |
| Asymptomatic HIV | 0.75 | 0.5–1 | [[3](#_ENREF_3), [22](#_ENREF_22)] |
| Symptomatic HIV | 0.15 | 0–0.3 | [[3](#_ENREF_3), [22](#_ENREF_22)] |
| AIDS | 0.10 | 0–0.2 | [[3](#_ENREF_3), [22](#_ENREF_22)] |
| **Annual entry and exit rates** |  |  |  |
| Entry to population | 0.030 | 0.025–0.031 | [[23](#_ENREF_23)] |
| Percentage of new entrants who are IDUs | 2.0% | 1.5%–2.0% | [[16](#_ENREF_16)] |
| Maturation | 0.029 | 0.028–0.034 | [[23](#_ENREF_23)] |
| Non-AIDS death rate, non-IDUs | 0.005 | 0.003–0.007 | [[24](#_ENREF_24)] |
| Non-AIDS death rate, IDUs not on MMT | 0.035 | 0.02–0.05 | [[11](#_ENREF_11), [25](#_ENREF_25)] |
| Non-AIDS death rate, IDUs on MMT | 0.015 | 0.009–0.021 | [[11](#_ENREF_11), [25](#_ENREF_25)] |
| Rate of spontaneous IDU quitting | 0.01 | 0.005–0.015 | [[1](#_ENREF_1), [10](#_ENREF_10), [11](#_ENREF_11), [26](#_ENREF_26)] |
| Rate of starting injection drug use | 0.0003 | 0.0002–0.0004 | Estimated [[1](#_ENREF_1), [10](#_ENREF_10), [11](#_ENREF_11), [26](#_ENREF_26)] |
| **Annual HIV progression rates** |  |  |  |
| AIDS death rate, no ART | 0.517 | 0.4–0.6 | [[22](#_ENREF_22), [27-29](#_ENREF_27)] |
| AIDS death rate, ART | 0.416 | 0.3–0.5 | [[22](#_ENREF_22), [27-29](#_ENREF_27)] |
| Progression rate asymptomatic to symptomatic | 0.136 | 0.10–0.15 | [[22](#_ENREF_22)] |
| Progression rate symptomatic to AIDS, no ART | 0.395 | 0.3–0.5 | [[22](#_ENREF_22)] |
| Progression rate symptomatic to AIDS, ART | 0.062 | 0.04–0.08 | [[22](#_ENREF_22)] |
| **Antiretroviral therapy (ART)** |  |  |  |
| Access to ART – eligible non-IDUs | 22% | 7%–11% | [[30](#_ENREF_30)] |
| Access to ART – eligible IDUs | 2% | 0%–5% | Estimated [[6](#_ENREF_6), [12](#_ENREF_12), [31](#_ENREF_31)] |
| Access to ART – eligible IDUs on MMT | 25% | 0%–30% | Estimated [[13](#_ENREF_13), [15](#_ENREF_15)] |
| Annual rate of quitting ART – non-IDUs | 0.125 | 0.05–0.5 | Calculated [[17](#_ENREF_17)] |
| Annual rate of quitting ART – IDUs not on MMT | 0.65 | 0.40–0.90 | Estimated [[13](#_ENREF_13), [15](#_ENREF_15)] |
| Annual rate of quitting ART – IDUs on MMT | 0.40 | 0.25–0.65 | Estimated [[13](#_ENREF_13), [15](#_ENREF_15)] |
| Sexual transmission reduction if on ART | 96% | 50%–99% | [[3](#_ENREF_3), [22](#_ENREF_22), [32](#_ENREF_32), [33](#_ENREF_33)] |
| Injection equipment sharing transmission reduction if on ART | 50% | 10%–90% | Estimated [[1](#_ENREF_1), [3](#_ENREF_3), [33](#_ENREF_33)] |
| **Methadone maintenance treatment (MMT)** |  |  |  |
| Percent decrease in injection equipment sharing if on MMT | 85% | 60%–99% | [[10](#_ENREF_10), [11](#_ENREF_11), [13-15](#_ENREF_13)] |
| MMT retention, 6 months | 75% | 50%–90% | [[13](#_ENREF_13), [15](#_ENREF_15)] |
| Percentage MMT “graduation” | 5% | 1%–7% | [[13](#_ENREF_13), [15](#_ENREF_15)] |
| **Pre-exposure prophylaxis (PrEP)** |  |  |  |
| Percent change in risky injections due to PrEP | 0% | -20%–20% | [[34-36](#_ENREF_34)] |
| Percent change in risky sexual contacts due to PrEP | 0% | -20%–20% | [[34-36](#_ENREF_34)] |
| Sexual transmission reduction if on PrEP | 49% | 10%–72% | [[37](#_ENREF_37)] |
| Needle sharing transmission reduction if on PrEP | 49% | 10%–72% | [[37](#_ENREF_37)] |
| **Injection behavior** |  |  |  |
| Number of injections per year | 250 | 200–300 | [[1](#_ENREF_1), [3](#_ENREF_3), [6-8](#_ENREF_6), [12](#_ENREF_12)] |
| Percent of injections that use shared equipment | 25% | 10%–40% | [[1](#_ENREF_1), [3](#_ENREF_3), [6-8](#_ENREF_6), [12](#_ENREF_12)] |
| Probability of transmission per infected contact – no ART, no PrEP | 0.005 | 0.0025–0.01 | [[8](#_ENREF_8)] |
| **Sexual behavior** |  |  |  |
| Number of sexual partners per year – IDUs | 4.3 | 1.5–4.5 | [[1](#_ENREF_1), [3](#_ENREF_3), [8](#_ENREF_8)] |
| Number of sexual partners per year – non-IDUs | 1.3 | 1–1.8 | [[1](#_ENREF_1), [3](#_ENREF_3), [8](#_ENREF_8)] |
| Percentage of IDU sexual contacts with other IDUs | 45% | 20%–70% | [[3](#_ENREF_3), [6-8](#_ENREF_6), [12](#_ENREF_12)] |
| Condom usage rate – IDUs not on MMT or PrEP | 40% | 20%–60% | [[1](#_ENREF_1), [3](#_ENREF_3), [6-8](#_ENREF_6)] |
| Condom usage rate – IDUs not on MMT but on PrEP | 40% | 20%–60% | Estimated |
| Condom usage rate – IDUs on MMT but not PrEP | 45% | 25%–65% | [[1](#_ENREF_1), [3](#_ENREF_3), [6-8](#_ENREF_6)] |
| Condom usage rate – IDUs on MMT and PrEP | 45% | 25%–65% | Estimated |
| Condom usage rate – non-IDUs | 45% | 30%–70% | [[1](#_ENREF_1), [3](#_ENREF_3), [6-8](#_ENREF_6)] |
| Condom effectiveness | 90% | 85%–95% | [[3](#_ENREF_3), [9-11](#_ENREF_9)] |
| Sexual transmission reduction if on ART | 96% | 50%–99% | [[3](#_ENREF_3), [22](#_ENREF_22), [32](#_ENREF_32), [33](#_ENREF_33)] |
| **Chance of transmitting HIV per sexual partnership, no ART, no PrEP** |  |  |  |
| Early HIV | 0.04 | 0.01–0.05 | Estimated [[3](#_ENREF_3), [8](#_ENREF_8)] |
| Late HIV, no ART | 0.05 | 0.02–0.07 | Estimated [[3](#_ENREF_3), [8](#_ENREF_8)] |
| AIDS, no ART | 0.08 | 0.05–0.11 | Estimated [[3](#_ENREF_3), [8](#_ENREF_8)] |
| **Quality adjustments** |  |  |  |
| IDU, no MMT, no HIV | 0.90 | 0.8–1.0 | [[3](#_ENREF_3), [10](#_ENREF_10), [11](#_ENREF_11), [22](#_ENREF_22), [38](#_ENREF_38)] |
| IDU, no MMT, early HIV | 0.85 | 0.75–1.0 | [[3](#_ENREF_3), [10](#_ENREF_10), [11](#_ENREF_11), [22](#_ENREF_22), [38](#_ENREF_38)] |
| IDU, no MMT, late HIV | 0.73 | 0.65–0.77 | [[3](#_ENREF_3), [10](#_ENREF_10), [11](#_ENREF_11), [22](#_ENREF_22), [38](#_ENREF_38)] |
| IDU, no MMT, AIDS | 0.63 | 0.56–0.72 | [[3](#_ENREF_3), [10](#_ENREF_10), [11](#_ENREF_11), [22](#_ENREF_22), [38](#_ENREF_38)] |
| IDU, no MMT, PrEP | 0.90 | 0.8–1.0 | Estimated |
| MMT, no HIV | 0.95 | 0.84–1.00 | Calculated |
| MMT, early HIV | 0.90 | 0.75–1.00 | Calculated |
| MMT, late HIV | 0.77 | 0.65–0.80 | Calculated |
| MMT, AIDS | 0.67 | 0.57–0.75 | Calculated |
| MMT, PrEP | 0.95 | 0.84–1.00 | Estimated |
| Non-IDU, no HIV | 1 | 0.9–1.0 | [[3](#_ENREF_3), [10](#_ENREF_10), [11](#_ENREF_11), [22](#_ENREF_22), [38](#_ENREF_38)] |
| Non-IDU, early HIV | 0.94 | 0.85–1.0 | [[3](#_ENREF_3), [10](#_ENREF_10), [11](#_ENREF_11), [22](#_ENREF_22), [38](#_ENREF_38)] |
| Non-IDU, late HIV | 0.81 | 0.70–0.90 | [[3](#_ENREF_3), [10](#_ENREF_10), [11](#_ENREF_11), [22](#_ENREF_22), [38](#_ENREF_38)] |
| Non-IDU, AIDS | 0.70 | 0.60–0.80 | [[3](#_ENREF_3), [10](#_ENREF_10), [11](#_ENREF_11), [22](#_ENREF_22), [38](#_ENREF_38)] |
| Percentage increase if on MMT | 50% | 0.3–0.7 | [[10](#_ENREF_10), [14](#_ENREF_14), [39-41](#_ENREF_39)] |
| Percentage increase if on ART | 10% | 0.0–0.3 | [[22](#_ENREF_22), [42-45](#_ENREF_42)] |
| **Annual costs (US$)** |  |  |  |
| Non-HIV medical care | 311 | 200–450 | [[46](#_ENREF_46)] |
| HIV care | 1200 | 800–1600 | Estimated [[17](#_ENREF_17)] |
| ART – IDUs not on MMT (including IDU services) | 950 | 750–2500 | [[1](#_ENREF_1), [3](#_ENREF_3), [24](#_ENREF_24), [47](#_ENREF_47), [48](#_ENREF_48)] |
| ART – IDUs on MMT (including IDU services) | 750 | 550–2300 | [[1](#_ENREF_1), [3](#_ENREF_3), [24](#_ENREF_24), [47](#_ENREF_47), [48](#_ENREF_48)] |
| ART – non-IDUs | 450 | 250–2000 | [[1](#_ENREF_1), [3](#_ENREF_3), [24](#_ENREF_24), [47](#_ENREF_47), [48](#_ENREF_48)] |
| MMT (including counseling services) | 368 | 200–500 | [[1](#_ENREF_1), [3](#_ENREF_3), [49](#_ENREF_49)] |
| PrEP (including counseling services) | 950 | 100–1500 | Estimated |
| **Discount rate** | 3% | 0%–5% | [[47](#_ENREF_47)] |

IDU = injection drug user, ART = antiretroviral therapy, MMT = methadone maintenance treatment, PrEP = pre-exposure prophylaxis

**Table S3. Initial population distribution for the model.***

| **Population Group** | **Uninfected** | **Early HIV** | **Late HIV, Untreated** | **AIDS, Untreated** | **Late HIV,**  **On ART** | **AIDS,**  **On ART** |
| --- | --- | --- | --- | --- | --- | --- |
| IDUs | 9,408 (0.941%) | 4,944 (0.494%) | 969 (0.097%) | 646 (0.065%) | 20 (0.0020%) | 13 (0.0013%) |
| General population | 974,292 (97.43%) | 7,281 (0.728%) | 1,136 (0.114%) | 757 (0.076%) | 320 (0.032%) | 214 (0.021%) |

*Distribution of a population of 1,000,000 individuals: 1.6% of the total population is an injection drug user (IDU); 75% of infected individuals are in the early HIV infection stage, 15% in the late stage, and 10% have AIDS; 22% of eligible non-IDUs and 2% of eligible IDUs are on antiretroviral therapy (ART).

**Table S4. HIV infections averted: results of one-way sensitivity analyses**.

|  | **Single Interventions** | | | | **Dual Interventions** | | | | | **All Interventions** | |
| --- | --- | --- | --- | --- | --- | --- | --- | --- | --- | --- | --- |
| **Parameter** | **25% PrEP** | **50% PrEP** | **ART** | **MMT** | **MMT, 25% PrEP** | **MMT, 50% PrEP** | **MMT, ART** | **ART, 25% PrEP** | **ART, 50% PrEP** | **MMT, ART, 25% PrEP** | **MMT, ART, 50% PrEP** |
| Base case values | 3552 | 5464 | 3935 | 4723 | 9130 | 11,072 | 8164 | 7548 | 9401 | 12,453 | 14,267 |
| Effectiveness of PrEP in reducing HIV acquisition |  |  |  |  |  |  |  |  |  |  |  |
| Low value (10%) | 509 | 752 | 3935 | 4723 | 5425 | 5719 | 8164 | 4466 | 4712 | 8866 | 9152 |
| High value (72%) | 6598 | 10,213 | 3935 | 4723 | 12,088 | 15,177 | 8164 | 10,454 | 13,805 | 15,188 | 17,992 |
| Percent decrease in injection equipment sharing if on MMT |  |  |  |  |  |  |  |  |  |  |  |
| Low value (60%) | 3552 | 5464 | 3935 | 3258 | 7530 | 9512 | 6744 | 7548 | 9401 | 10,894 | 12,855 |
| High value (99%) | 3552 | 5464 | 3935 | 5631 | 10,067 | 11,965 | 9018 | 7548 | 9401 | 13,306 | 15,071 |
| Percentage of sexual contacts shared by IDUs with IDUs |  |  |  |  |  |  |  |  |  |  |  |
| Low value (20%) | 4462 | 6745 | 4287 | 6272 | 11,371 | 13,481 | 10,258 | 9182 | 11,317 | 15,069 | 16,998 |
| High value (70%) | 2732 | 4267 | 2912 | 3400 | 7013 | 8703 | 6095 | 5821 | 7376 | 9755 | 11,387 |
| Effectiveness of ART in reducing HIV transmission via needlesharing |  |  |  |  |  |  |  |  |  |  |  |
| Low value (10%) | 3542 | 5450 | 3140 | 4572 | 8959 | 10,904 | 7165 | 6498 | 8276 | 11,327 | 13,155 |
| High value (90%) | 3563 | 5478 | 4856 | 4878 | 9303 | 11,241 | 9297 | 8733 | 10,652 | 13,671 | 15,447 |

PrEP = oral pre-exposure prophylaxis for injection drug users (IDUs); ART = antiretroviral therapy for 80% of eligible individuals; MMT = methadone maintenance treatment for 25% of IDUs; 25% PrEP = PrEP for 25% of uninfected IDUs; 50% PrEP = PrEP for 50% of uninfected IDUs

**REFERENCES**

1. Alistar SS, Owens DK and Brandeau ML (2011) Effectiveness and cost effectiveness of expanding harm reduction and antiretroviral therapy in a mixed HIV epidemic: A modeling analysis for Ukraine. PLoS Med 8: e1000423.
2. Sanders GD, Bayoumi AM, Sundaram V, Bilir SP, Neukermans CP, et al. (2005) Cost effectiveness of screening for HIV in the era of highly active antiretroviral therapy. New Engl J Med 352: 32-47.
3. Long EF, Brandeau ML, Galvin CM, Vinichenko T, Tole SP, et al. (2006) Effectiveness and cost-effectiveness of strategies to expand antiretroviral therapy in St. Petersburg, Russia. AIDS 20: 2207-2215.
4. Smyrnov P (2007) Making the change: 3 years of Global Fund supported treatment in Ukraine - Evidence versus policy. Tbilisi, Georgia: International HIV/AIDS Alliance in Ukraine.
5. The World Bank (2006) Socioeconomic Impact of HIV/AIDS in Ukraine. Washington, DC: World Bank.
6. Booth RE, Kwiatkowski CF, Brewster JT, Sinitsyna L and Dvoryak S (2006) Predictors of HIV sero-status among drug injectors at three Ukraine sites. AIDS 20: 2217-2223.
7. Booth RE, Kwiatkowski CF, Mikulich-Gilbertson SK, Brewster JT, Salomonsen-Sautel S, et al. (2006) Predictors of risky needle use following interventions with injection drug users in Ukraine. Drug Alcohol Depend 82: S49-S55.
8. Vickerman P, Kumaranayake L, Balakireva O, Guinness L, Artyukh O, et al. (2006) The cost-effectiveness of expanding harm reduction activities for injecting drug users in Odessa, Ukraine. Sex Transm Dis 33: S89-S102.
9. Pinkerton SD and Abramson PR (1997) Effectiveness of condoms in preventing HIV transmission. Soc Sci Med 44: 1303-1312.
10. Zaric GS, Barnett PG and Brandeau ML (2000) HIV transmission and the cost-effectiveness of methadone maintenance. Am J Public Health 90: 1100-1111.
11. Zaric GS, Brandeau ML and Barnett PG (2000) Methadone maintenance and HIV prevention: a cost-effectiveness analysis. Manage Sci 46: 1013-1031.
12. Barcal K, Schumacher J, Dumchev K and Moroz L (2005) A situational picture of HIV/AIDS and injection drug use in Vinnitsya, Ukraine. Harm Reduct J 2: 16.
13. Bruce RD, Dvoryak S, Sylla L and Altice FL (2007) HIV treatment access and scale-up for delivery of opiate substitution therapy with buprenorphine for IDUs in Ukraine--programme description and policy implications. Int J Drug Policy 18: 326-328.
14. Connock M, Juarez-Garcia A, Jowett S, Frew E, Liu Z, et al. (2007) Methadone and buprenorphine for the management of opioid dependence: a systematic review and economic evaluation. Health Technology Assessment. Southampton, United Kingdom: National Institute for Health Research - Health Technology Assessment Programme.
15. Dvoriak S (2007) Opioid Substitution Therapy (OST) with Buprenorphine in Ukraine - Way to Prevent HIV/AIDS among IDUs. 4th IAS Conference on HIV Pathogenesis, Treatment and Prevention. Sydney, Australia.
16. Kruglov YV, Kobyshcha YV, Salyuk T, Varetska O, Shakarishvili A, et al. (2008) The most severe HIV epidemic in Europe: Ukraine's national HIV prevalence estimates for 2007. Sex Transm Infect 84: i37-i41.
17. Joint United Nations Programme on HIV/AIDS (UNAIDS) (2008) Ukraine - National report on monitoring progress towards the UNGASS declaration of commitment on HIV/AIDS. Geneva: United Nations.
18. Celentano DD, Beyrer C, Wolfe D, Elovich R, Boltaev A, et al. (2008) HIV in Central Asia: Tajikistan, Uzbekistan and Kyrgyzstan. Public Health Aspects of HIV/AIDS in Low and Middle Income Countries. Springer New York. pp. 557-581.
19. Mathers BM, Degenhardt L, Phillips B, Wiessing L, Hickman M, et al. (2008) Global epidemiology of injecting drug use and HIV among people who inject drugs: a systematic review. Lancet 372: 1733-1745.
20. Ministry of Health of Ukraine (2012) Ukraine Harmonized AIDS Response Progress Report. Kyev, Ukraine: Ministry of Health Ukraine.
21. Joint United Nations Programme on HIV/AIDS (UNAIDS) (2008) Ukraine - Country situation fact sheet. Geneva: United Nations.
22. Sanders GD, Bayoumi AM, Sundaram V, Bilir SP, Neukermans CP, et al. (2005) Cost-effectiveness of screening for HIV in the era of highly active antiretroviral therapy. N Engl J Med 352: 570-585.
23. Feshbach M and Galvin C (2005) HIV/AIDS in Ukraine - An Analysis of Statistics. Washington, DC: Woodrow Wilson International Center for Scholars.
24. World Health Organization (WHO) (2005) Summary Country Profile for HIV/AIDS Treatment Scale-Up - Ukraine. Geneva: World Health Organization.
25. Grönbladh L, Öhlund LS and Gunne LM (1990) Mortality in heroin addiction: impact of methadone treatment. Acta Psychiatr Scand 82: 223-227.
26. Grönbladh L and Gunne LM (1989) Methadone-assisted rehabilitation of Swedish heroin addicts. Drug and Alcohol Dependence 24: 31-37.
27. Egger M, May M, Chêne G, Phillips AN, Ledergerber B, et al. (2002) Prognosis of HIV-1-infected patients starting highly active antiretroviral therapy: a collaborative analysis of prospective studies. Lancet 360: 119-129.
28. Grabar S, Moing VL, Goujard C, Leport C, Kazatchkine MD, et al. (2000) Clinical outcome of patients with HIV-1 infection according to immunologic and virologic response after 6 months of highly active antiretroviral therapy. Ann Intern Med 133: 401-410.
29. Wood E, Hogg RS, Yip B, Harrigan PR, O'Shaughnessy MV, et al. (2003) Effect of medication adherence on survival of HIV-infected adults who start highly active antiretroviral therapy when the CD4+ cell count is 0.200 to 0.350 x 109 cells/L. Ann Intern Med 139: 810-816.
30. World Health Organization (2013) Data on the HIV/AIDS response: Antiretroviral therapy coverage by country. Geneva, Switzerland.
31. Wolfe D, Carrieri MP and Shepard D (2010) Treatment and care for injecting drug users with HIV infection: a review of barriers and ways forward. Lancet 376: 355-366.
32. Castilla J, del Romero J, Hernando V, Marincovich B, Garcia S, et al. (2005) Effectiveness of highly active antiretroviral therapy in reducing heterosexual transmission of HIV. J AIDS 40: 96-101.
33. Cohen MS, Chen YQ, McCauley M, Gamble T, Hosseinipour MC, et al. (2011) Prevention of HIV-1 infection with early antiretroviral therapy. N Engl J Med 365: 493-505.
34. Golub SA, Kowalczyk W, Weinberger CL and Parsons JT (2010) Preexposure prophylaxis and predicted condom use among high-risk men who have sex with men. J Acquir Immune Defic Syndr 54: 548-555.
35. Golub SA, Operario D and Gorbach PM (2010) Pre-exposure prophylaxis state of the science: empirical analogies for research and implementation. Curr HIV/AIDS Rep 7: 201-209.
36. Myers GM and Mayer KH (2011) Oral preexposure anti-HIV prophylaxis for high-risk U.S. populations: current considerations in light of new findings. AIDS Patient Care STDS 25: 63-71.
37. Choopanya K, Martin M, Suntharasamai P, Sangkum U, Mock PA, et al. (2013) Antiretroviral prophylaxis for HIV infection among people who inject drugs in Bangkok, Thailand: a randomized, double-blind, placebo-controlled trial. Lancet 381: 2083-2090.
38. Tengs TO and Lin TH (2002) A meta-analysis of utility estimates for HIV/AIDS. Med Decis Making 22: 475-481.
39. Lucas GM, Mullen BA, Weidle PJ, Hader S, McCaul ME, et al. (2006) Directly administered antiretroviral therapy in methadone clinics is associated with improved HIV treatment outcomes, compared with outcomes among concurrent comparison groups. Clinical Infectious Diseases 42: 1628-1635.
40. Marsch LA (1998) The efficacy of methadone maintenance interventions in reducing illicit opiate use, HIV risk behavior and criminality: a meta-analysis. Addiction 93: 515-532.
41. Schilling R, Dornig K and Lungren L (2006) Treatment of heroin dependence: effectiveness, costs, and benefits of methadone maintenance. Res Social Work Practice 16: 48-56.
42. Gill CJ, Griffith JL, Jacobson D, Skinner S, Gorbach SL, et al. (2002) Relationship of HIV viral loads, CD4 counts, and HAART use to health-related quality of life. J AIDS 30: 485-492.
43. Jelsma J, MacLean E, Hughes J, Tinise X and Darder M (2005) An investigation into the health-related quality of life of individuals living with HIV who are receiving HAART. AIDS Care 17: 579 - 588.
44. Liu C, Ostrow D, Detels R, Hu Z, Johnson L, et al. (2006) Impacts of HIV infection and HAART use on quality of life. Qual Life Res 15: 941-949.
45. Mannheimer SB, Matts J, Telzak E, Chesney M, Child C, et al. (2005) Quality of life in HIV-infected individuals receiving antiretroviral therapy is related to adherence. AIDS Care 17: 10-22.
46. UNAIDS/WHO Working Group on Global HIV/AIDS and STI Surveillance (2008) Epidemiological Factsheet on HIV and AIDS: Core Data on Epidemiology and Response - Ukraine. Geneva: World Health Organization (WHO) and Joint United Nations Programme on HIV/AIDS (UNAIDS).
47. World Health Organization (WHO) (2003) Making Choices in Health: WHO Guide to Cost-Effectiveness Analysis. Geneva: World Health Organization.
48. World Health Organization (WHO) (2009) Global Price Reporting Mechanism. Geneva: World Health Organization.
49. International HIV/AIDS Alliance in Ukraine (2008) Methadone substitution therapy starts in Ukraine. Kyiv, Ukraine: International HIV/AIDS Alliance in Ukraine.
